# Prognostic implications of surgical specimen quality on the oncological outcomes of open and laparoscopic surgery in mid and low rectal cancer

**DOI:** 10.1007/s00423-021-02351-1

**Published:** 2021-10-30

**Authors:** Vicente Pla-Martí, José Martín-Arévalo, David Moro-Valdezate, Stephanie García-Botello, Leticia Pérez-Santiago, María Lapeña-Rodríguez, Mireia Bauzá-Collado, Marisol Huerta, Susana Roselló-Keränen, Alejandro Espí-Macías

**Affiliations:** 1 Colorectal Surgery Unit, Department of General and Digestive Surgery, Biomedical Research Institute INCLIVA, Hospital Clínico Universitario, Av. Blasco Ibáñez, 17. 46010, Valencia, Spain; 2 Department of Surgery, Universidad de Valencia, Valencia, Spain; 3 Department of Medical Oncology, INCLIVA Biomedical Research Institute, University of Valencia, Valencia, Spain; 4 Instituto de Salud Carlos III, CIBERONC, Valencia, Spain

**Keywords:** Rectal neoplasms, Laparoscopy, Colorectal surgery, Survival

## Abstract

**Purpose:**

Determine differences in pathologic outcomes between laparoscopic (LAP) and open surgery (OPEN) for mid and low rectal cancer and its influence in long-term oncological outcomes.

**Methods:**

Retrospective case matched study at a tertiary institution. Adults with rectal cancer below 12 cm from the anal verge operated between January 2005 and September 2018 were included. Primary outcomes were quality of specimen, overall survival (OS), disease-free survival (DFS), and local recurrence (LR).

**Results:**

The study included 311 patients, LAP = 108 (34.7%), OPEN = 203 (65,3%). A successful resection was accomplished in 81% of the LAP group and in 84.5% of the OPEN (*p* = 0.505). No differences in free distal margin (LAP = 100%, OPEN = 97.5%; *p* = 0.156) or circumferential resection margin (LAP = 95.2%, OPEN = 93.2%; *p* = 0.603) were observed. However, mesorectum quality was incomplete in 16.2% for LAP and in 8.1% for OPEN (*p* = 0.048). OS was 91.1% for LAP and 81.1% for OPEN (*p* = 0.360). DFS was 81.4% for LAP and 77.5% for OPEN (*p* = 0.923). Overall, LR was 2.3% without differences between groups.

**Conclusions:**

Laparoscopic approach could affect the quality of surgical specimen due to technical aspects. However, if principles of surgical oncology are respected, minor pathologic differences in the quality of the mesorectum may not influence on the long-term oncologic outcomes.

## Introduction

Surgical resection remains the treatment of choice for rectal cancer. Outcomes have improved significantly in the last decades since Heald described the principles of total mesorectal resection [1]. This change in surgical technique allowed a decrease in local recurrence (LR) and functional results improvement. The multidisciplinary management has been another essential aspect that has improved the treatment of these patients. In this sense, the role of the pathologist auditing the quality of the surgical specimen is highly relevant. The quality of the mesorectum, the circumferential resection margin (CRM), and distal margin (DM) determine the oncological results such as overall survival (OS), disease free survival (DFS), and LR [2].

The evolution of laparoscopic surgical approach has allowed using minimally invasive techniques in an increasing number of complicated procedures. The laparoscopic approach offers several advantages such as early mobilization, shorter length of stay, earlier recovery of normal functions, less postoperative pain, and better cosmetic results [3, 4]. However, concerns about safety in oncological outcomes of laparoscopic approach remain in patients with rectal cancer, due to the technical complexity of surgery in the pelvis and the difficulties to control locally the tumor. Although several randomized controlled trials (RCTs) showed similar oncologic results in laparoscopy compared with open surgery [5–7], the two most recent RCTs [8, 9] and a systematic review found contradictory results and placed previous conclusions under debate [10].

The primary endpoint of this study was to assess the quality of the specimen in laparoscopic and open rectal cancer resections and its influence in long-term oncological outcomes (LR, OS, and DFS).

## Material and methods

An observational retrospective case matched study was carried out in patients with mid and lower third rectal cancer undergoing surgery with curative intent between January 2005 and December 2017. The pathological and the oncological outcomes of the laparoscopic surgery (LAP) were compared with those of the open approach (OPEN).

### Data source

A retrospective analysis of a prospective database was conducted. The complete medical history and the medical records of primary care of each patient were reviewed. The open cases were matched to laparoscopic cases by a propensity score analysis to obtain comparable groups of patients. The study was approved by the local Ethics Committee.

### Patients

The inclusion criteria were age over 18 years with adenocarcinoma of the rectum located by rigid proctoscopy at or below 12 cm from the anal verge, with stages I, II, and III. Exclusion criteria were the transanal surgical approach, patients who underwent palliative surgery, and clinical stage IV.

Neoadjuvant chemoradiation therapy was indicated in those patients at high risk of presenting LR (T4 or CRM ≤ 2 mm at MRI assessment). The surgical techniques were low anterior resection (LAR) and extralevator abdominoperineal resection (APR) following strictly the oncological principles of TME in all cases. Dedicated colorectal surgeons with more than 10 years of experience performed all surgeries.

### Outcome variables

The primary outcomes were quality of the specimen, OS, DFS, and LR at 5 years. Quality of the mesorectum was classified as follows: *Complete*, intact mesorectum with only minor irregularities of a smooth mesorectal surface. No defect is deeper than 5 mm, and there is no coning toward the distal margin of the specimen. There is a smooth circumferential resection margin on slicing; *Nearly-complete*, moderate bulk to the mesorectum, but irregularity of the mesorectal surface. At no site is the muscularis propria visible, with the exception of the insertion of the levator muscles; *or Incomplete*, little bulk to mesorectum with defects down onto muscularis propria and/or very irregular circumferential resection margin [11]. Specimen processing and assessment was performed by the same team of pathologists throughout the whole study period using a standardized procedure after proper training.

CRM was considered as positive when the distance between the surgical resection margin and the deepest cancer invasion was ≤ 1 mm. Lymph node yield, proximal, and DM were recorded. “*Successful resection*” was considered when surgical specimen showed negative CRM, negative DM (> 1 cm), and complete or near complete mesorectum. The follow-up protocol was made according to the current clinical guidelines. The patients with stage-III and those with stage-II at high risk of local or distal recurrence received postoperative chemotherapy (except contraindications due to comorbidity or serious postoperative complications that delayed recovery beyond 8 weeks).

LR was defined as recurrence within the pelvis and distal recurrence as recurrence outside the pelvis. OS was defined as time from surgery to death for any cause and DFS as time interval between treatment and the date of disease progression, death for any cause, or development of second primary cancer. The proof of life was based on the health affiliation records.

### Study variables

The variables analyzed were age, sex, body mass index (BMI), American Society of Anaesthesiologist (ASA) score, Charlson comorbidity index, clinical stage, neoadjuvant chemoradiotherapy, and surgical approach. Peri-operative data included blood transfusion, operative time, and length of stay. Pathological data included CRM, DM clearance, quality of the mesorectal excision, and lymph node harvest. Postoperative morbidity was recorded following Clavien-Dindo classification. Perioperative death was considered as fatal outcome within 30 postoperative days. The outcome variables included postoperative complications, mortality, successful resection, OS, DFS, and LR.

### Analysis

Descriptive analysis was driven for all the variables. Normality for quantitative variables was assessed with the Kolmogorov-Smirnoff test. Parametric and non-parametric tests were used based in normality of quantitative variable. Data were reported as median and range in non-normal quantitative variables and mean with standard deviation in otherwise. Five-year OS, DFS, and LR were analyzed using Kaplan–Meier with log rank test. OS and DFS were compared between both surgical approaches. Statistical significance for *p-*value was set at 0.05.

**Fig. 1:**
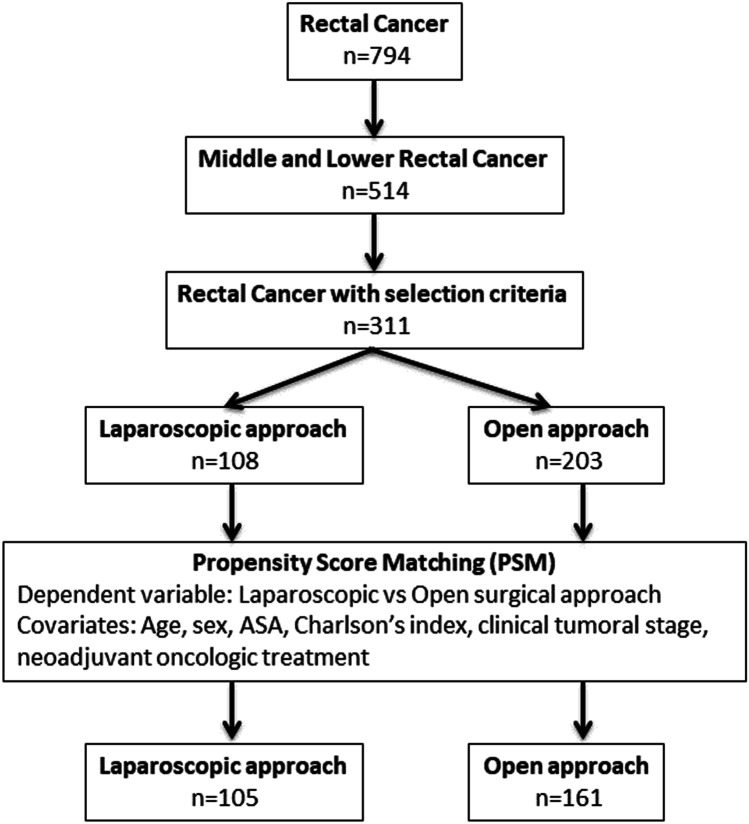
Flowchart of study population selection and matching by propensity score

 A propensity score matching was used to minimize potential selection bias. Two patients in the open approach were matched to each individual in the laparoscopic group. The confounding variables to calculate the propensity score were age, sex, ASA score, neoadjuvant treatment, Charlson comorbidity index, and oncologic stage. Caliber of 0.2 was used [12, 13] (Fig. 1).

Statistic software used was SPSS v25 (IBM Corp., Armonk, NY, USA) and R (3.2.0) for statistical analyses (R Foundation for Statistical Computing, Vienna, Austria, 2013). *PsMatching* script of R integrated in SPSS was used to assess the propensity score.

## Results

During the study period, 311 patients were included and underwent elective curative surgery. The minimum follow-up of the series was 3 years. There were two cases lost to follow-up due to change of district of residence. Clinical and demographic characteristics are summarized in Table 1. A total of 108 (34.7%) patients underwent LAP and 203 OPEN surgery. The median follow-up was 63 months (range, 164 months). Throughout the study, the LAP approach increased significantly (*p* < 0.001), with 80% of laparoscopic surgeries performed in the latter 5 years. Conversion to open surgery was necessary in 8 cases (7.41%) and these patients were assigned to the LAP group. All cases of conversions were due to difficulties during dissection of the distal rectum.

**Table 1:** Demographic, clinical, and histological characteristics of the patients (overall series and post-propensity score matching)

	Overall series			Propensity score matched pairs		
	OPEN 203 (65.27)	LAP 108 (34.73)	*p* value	OPEN 161 (60.53)	LAP 105 (39.47)	*p* value
Age (years)*	70 (48)	66 (51)	0.050	67 (47)	66 (51)	0.369
**Sex (male)**	131 (64,5)	74 (68,5)	0.531	108 (61.1)	73 (69.5)	0.689
**Charlson score**	5 (10)	5 (10)	0.567	5(10)	5(10)	0.792
**ASA score**			0.980			0.977
I	17 (8.4)	9 (8.3)		15 (9.3)	9 (8.6)	
II	106 (52.2)	56 (51.9)		85 (52.8)	54 (51.4)	
III	77 (37.9)	42 (38.9)		59 (36.6)	41 (39)	
IV	3 (1.5)	1 (0.9)		2 (1.2)	1 (1)	
**BMI (kg/m**^**2**^)*	26.48 (38.71)	25.37 (30.72)	0.115	26.17 (38.71)	25.43 (30.72)	0.118
**Site of tumor**			0.721			0.900
Middle rectum (7–11 cm)	94 (46.3)	53 (49.1)		76 (47.2)	51 (48.6)	
Distal rectum (0–6 cm)	109 (53.7)	55 (50.9)		85 (52.8)	54 (51.4)	
**cT stage**			0.266			0.472
T1	19 (9.4)	5 (4.6)		14 (8.7)	5 (4.8)	
T2	38 (18.7)	18 (16.7)		26 (16.1)	17 (16.2)	
T3	146 (71.9)	85 (78.7)		121 (71.2)	83 (79)	
**Clinical stage**			0.115			0.690
I	67 (33)	25 (23.1)		43 (26.7)	25 (23.8)	
II	36 (17.7)	17 (15.7)		30 (18.6)	17 (16.2)	
III	100 (49.3)	66 (61.1)		88 (54.7)	63 (60)	
**Primary anastomosis**	143 (70.4)	81 (75)	0.428	118 (73.3)	79 (75.2)	0.418
Operative time (minutes)*	195 (340)	240 (400)	** < *0.001***	205 (335)	240 (400)	***0.002***
**Diverting stoma**	133 (65.5)	47 (43.5)	** < *0.001***	109 (67.7)	47 (44.8)	** < *0.001***
**Preoperative blood transfusion**	23 (11.3)	9 (8.3)	0.441	18 (11.2)	9 (8.6)	0.540
**Postoperative blood transfusion**	35 (17.2)	13 (12)	0.252	27 (16.8)	13 (12.4)	0.382
Lymph nodes examined*	17 (54)	14.5 (64)	0.102	17 (54)	14 (64)	0.056
**Pathological stage**			0.409			0.640
0	13 (6.4)	11 (10.2)		12 (7.5)	11 (10.5)	
I	79 (38.9)	36 (33.3)		54 (33.5)	36 (34.3)	
II	48 (23.6)	28 (25.9)		43 (26.7)	27 (25.7)	
III	63 (31)	33 (30.5)		52 (32.3)	31 (29.6)	
**Neoadjuvant treatment**	74 (36.5)	59 (54.6)	***0.003***	72 (44.7)	57 (54.3)	0.134
**Postoperative Complications**	45 (22.2)	19 (17.6)	0.379	33 (20.5)	19 (18.1)	0.752
**Mortality < 30 days**	10 (4.9)	4 (3.7)	0.777	6 (3.7)	4 (3.8)	1
Hospital stay (days)*	9 (59)	7 (84)	***0.001***	9 (59)	7 (84)	***0.004***

Values in parentheses are percentages

*BMI* body mass index, *ASA* American Society of Anaesthesiology

^*^Median (range)

Baseline information between the two groups was similar except shorter operative time (*p* < 0.001), higher rate of diverting ileostomies (*p* < 0.001) (odds ratio, 1.383; 95% confidence interval 1.153–1.659), and a lower rate of neoadjuvant treatment in the OPEN than in the LAP group (*p* = 0.003) (OR 1.611; 95% CI 1.188–2.186). Overall morbidity was similar between groups. The length of stay of the LAP group was significantly shorter compared to the OPEN group (7 vs 9 days, *p* = 0.001).

There were no significant differences in the number of patients receiving adjuvant chemotherapy between the LAP group (49.1%) and the OPEN group (39.4%) (*p* = 0.118).

### Pathologic outcomes

Overall, a successful resection was accomplished in 82.3% of patients, 12.5% presented an incomplete mesorectal excision, DM was involved in 1.3% of the cases and positive CRM was observed in 6.8%. There were no significant differences in the pathological outcomes between LAP and OPEN surgery (Table 2).

**Table 2:** Quality of the surgical specimen

	Overall series			Propensity score matched pairs		
	OPEN 203 (65.27)	LAP 108 (34.73)	*p* value	OPEN 161 (60.53)	LAP 105 (39.47)	*p* value
Quality of mesorectal excision: incomplete	21 (10.3)	18 (16.7)	0.149	13 (8.1)	17 (16.2)	0.048
Positive distal margin	4 (2)	0	0.302	4 (2.5)	0	0.156
Positive circumferential resection margin	15 (7.4)	6 (5.6)	0.640	11 (6.8)	5 (4.8)	0.603
Successful resection	169 (83,3)	87 (80.6)	0.640	136 (84.5)	85 (81)	0.505

Values in parentheses are percentages

### Long-term outcomes

Five-year OS was 92.7% for LAP and 88.6% for OPEN (*p* = 0.29). Interestingly, in the analysis of staging groups, no differences between laparoscopic and open resection were observed in OS and DFS.

Five-year DFS was 88.6% for LAP and 81% for OPEN (*p* = 0.83). Overall, LR was 2.25%, development of distant metastasis was found in 15.43% of the patients, and 0.3% of them had peritoneal carcinomatosis, without statistical differences between LAP and OPEN (*p* = 0.27, *p* = 0.75, and *p* = 1.00, respectively).

### Outcomes after propensity score matching

After adjusting the cases through the propensity score matching, we created two new groups of patients: 161 cases OPEN vs 105 LAP (Fig. 1). Both groups were completely comparable after case matching (Table 1).

Successful resection was achieved in 84.5% of the patients in the OPEN group and in 81% in the LAP group (*p* = 0.51). No differences in positive DM or CRM were observed. However, the quality of the mesorectum was incomplete in 16.2% for the LAP group and 8.1% for the OPEN one (*p* = 0.048) (Table 2).

Fifty-one patients (48.6%) in the LAP group and 70 patients (43.5%) in the OPEN group received adjuvant chemotherapy. This difference was not statistically significative (*p* = 0.451).

**Fig. 2:**
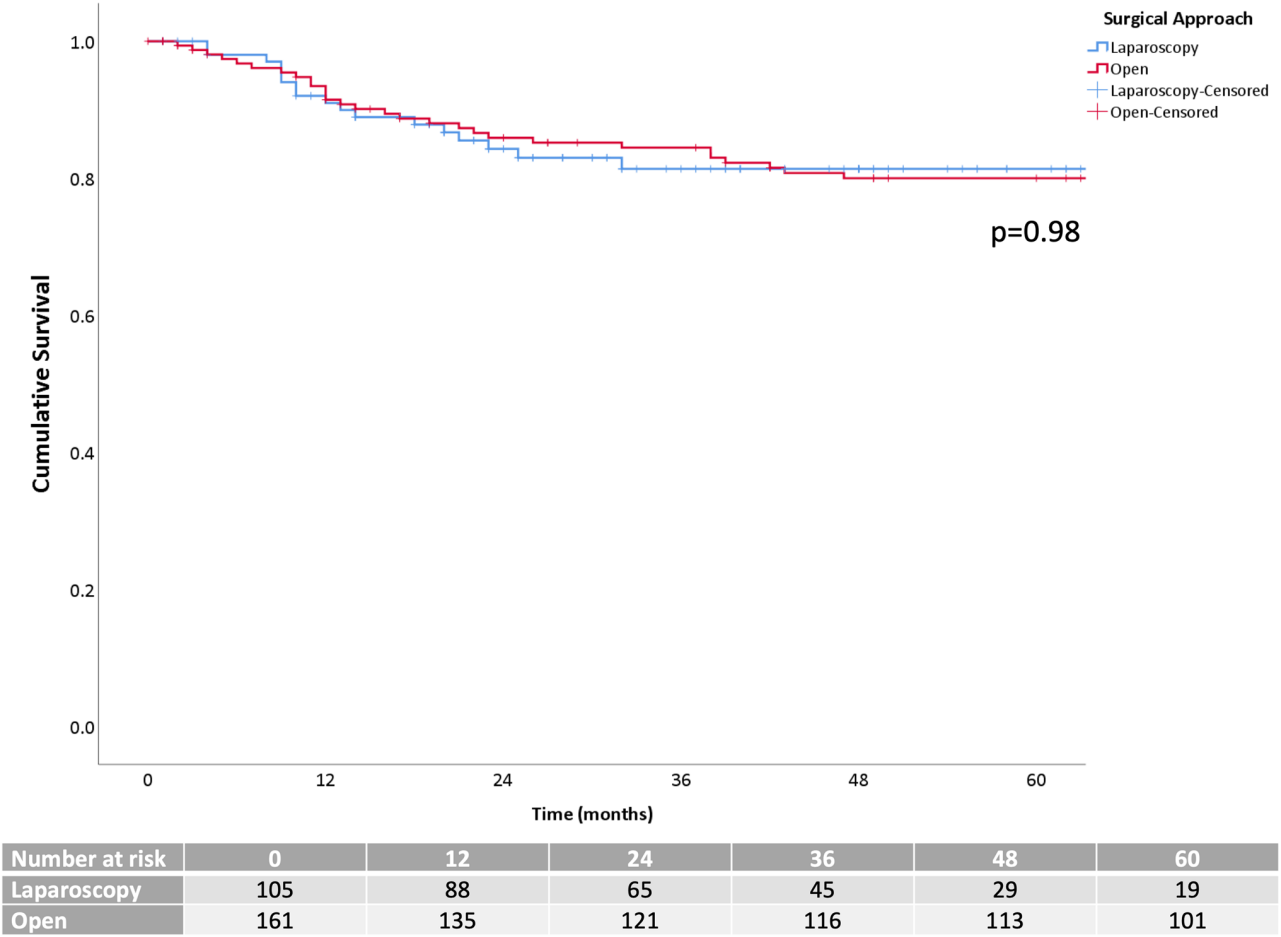
Kaplan–Meier curves of the overall survival after matching cases

**Fig. 3:**
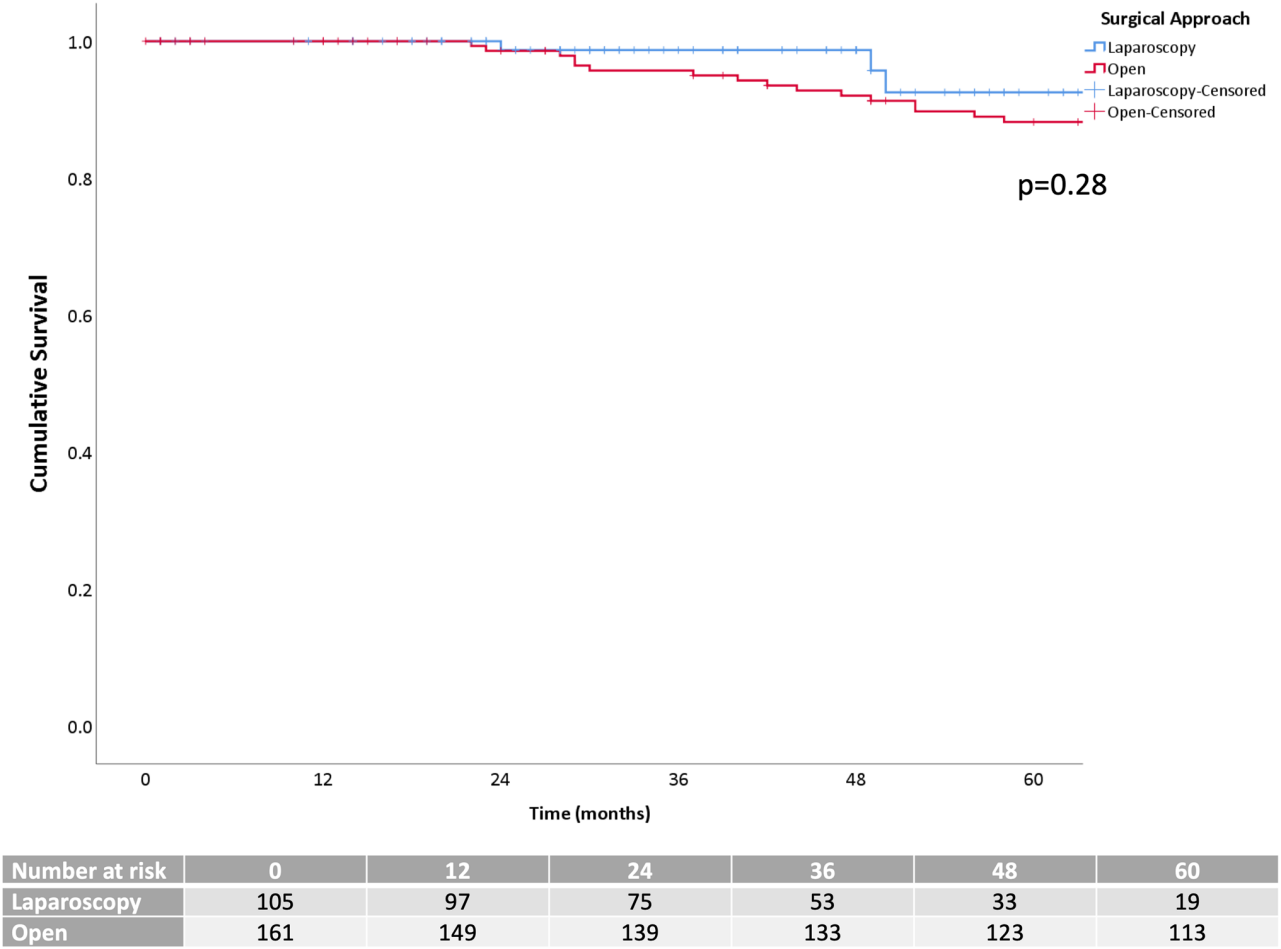
Kaplan–Meier curves of the disease-free survival after matching cases

Five-year OS was 92.5% in the LAP group and 88.2% in the OPEN one (*p* = 0.28) (Fig. 2). Five-year DFS was 81.4% for laparoscopic and 80% for open surgery (*p* = 0.98) (Fig. 3). LR was 2.3%, systemic recurrence was found in 16.2% of the patients, and peritoneal carcinomatosis in 0.4% of them without statistical differences between LAP and OPEN (*p* = 0.41, *p* = 1.00, and *p* = 1.00, respectively).

## Discussion

The results of the present study advocated that in unselected patients with stage I–III mid or low rectal cancer LAP was associated with similar oncological outcomes than OPEN resection. The first randomized trials (CLASICC, COLOR II, and COREAN) comparing laparoscopic with open rectal resection obtained similar results. In these studies, no differences were found in the quality of the mesorectum or in the DM or CRM between both groups. Short- and long-term outcomes showed no differences in morbidity, OS, DFS, or LR [3–7, 14, 15]. Consequently, for treatment of rectal cancer, laparoscopic approach seemed to be as safe as open surgery, with short-term benefits or even less morbidity [16]. Therefore, its use has increased in recent years. In our experience the laparoscopic modality has become the approach of choice since 2013, and it have been used in 80% of the cases in the last 5 years.

More recent phase III trials have questioned again the noninferiority of laparoscopic vs open surgery in the treatment of rectal cancer. ACOSOG Z6051 study included 486 patients with stage II–III tumors located at 12 cm or less above the anal margin who underwent surgery after neoadjuvant treatment. Satisfactory resection was accomplished in 82% of the patients operated on a minimally invasive approach vs 87% of those operated on with open surgery [8]. Similarly, in the Australian study ALaCaRT, which included 475 patients, the laparoscopic group showed 82% of successful resections vs 89% in the open surgery group [9]. The definition of a satisfactory surgical result in both studies was determined by the surgical specimen quality: completely satisfactory mesorectum, a clear DM, and a clear CRM. Both studies had high technical quality of the surgery, as shown by the low conversion rate, the high sphincter-sparing surgery rate, and the few general or anastomotic complications. The use of laparoscopic surgery compared with open did not meet the condition for noninferiority for pathologic outcomes in both studies; consequently, these results did not show enough evidence to recommend the use of laparoscopic resection routinely.

However, the subsequently oncologic results published from these studies were similar for both surgical approaches. Two-year DFS and LR results from ACOSOG Z6051 trial have been recently reported [17]. Differences between the LAP and the OPEN group in DFS (79.5 vs 83.2%), LR (2.1 vs 1.8%), and distant recurrence (14.6 vs 16.7%) were not found. Similarly, 2-year follow-up outcomes of the ALaCart trial comparing laparoscopic and open surgery for rectal cancer concluded that there were no outstanding differences in LR, DFS, and OS rates [18].

In our series, after adjusting by the propensity score, significant differences in successful resection rates between the two groups were not observed. Quality of the mesorectum was significantly inferior in the laparoscopic group although this difference did not affect the oncological outcomes since the rates of DFS and LR were similar between groups. ALaCaRT trial demonstrated that the lack of successful pathological resection was associated with poorer DFS and was mainly due to an involved CRM. The effect of laparoscopic surgery in LR or OS vanished after adjusting for positive CRM that seemed the better alternative of successful surgery for longer-term results [18]. ACOSOG Z6051 trial also found after multivariable analysis that the most important factor for an unsuccessful surgery was the positive CRM [17].

It has been stated that both poor mesorectum quality and positive CRM involve a reduced OS and increased LR. There is no doubt that oncological principles must be respected to perform a TME following the anatomical dissection planes to achieve an intact mesorectum. However, when expert surgeons perform laparoscopic rectal resection, obtaining lower rates of complete TME, it is usually due to small lesions in the fascia during the traction maneuvers required to complete the laparoscopic distal dissection and not because of having performed the dissection through an incorrect plane, therefore, may not threat seriously the prognosis of the patients, especially if the specimen is analyzed by dedicated pathologists meticulously following strict protocols, as in our case. High-quality surgical technique may also yield positive CRM due to the local spread of tumor cells, involving worse LR and DFS outcomes thus being wrongly assigned to the quality of technique. In the risk model, proposed in one study that assessed and identified predictors of CRM involvement for rectal cancer, the open procedure was an independent risk factor, with a rate of positive CRM of 10.0% compared with 3.9% for the laparoscopic approach (*p* < 0.001) [19]. In a recent Danish study, positive CRM was reported more frequently after open resection (6.3 vs 4.7%; *p* = 0.047). Nevertheless, both a multivariate and a propensity score-matched analysis were not able to demonstrate increased risk of positive CRM after laparoscopic vs open rectal resection [20]. These results were similar to those of the present study, since no differences in the positive CRM between the two groups were found (6.8 OPEN vs 4.8% LAP), and for this reason, no differences in LR or DFS were observed despite the worse quality of the TME in the laparoscopic group. As previously mentioned, these lesions could be developed during the traction and removal maneuvers of the surgical specimen, since in all cases, the embryological plane was respected during dissection, and should not be understood as an incorrect resection plane.

In challenging cases (bulky tumors, lower third location, narrow pelvis, or obese patients), the risk of obtaining a worse pathological outcome is greater. None of the RCTs previously mentioned, nor the present study, analyzed the differences in the pathological results in subgroups of patients with different technical difficulties. Robotic surgery, transanal total mesorectal excision (TaTME), and the design of new instruments could improve surgical specimen quality with good oncological results and allow the use of minimally invasive techniques even in difficult cases.

In the present series, before matching, patients operated on by laparoscopy had better oncological outcomes than those operated by open procedure with differences of 7.6% in DFS and 4.1% in OS. After matching, the differences decreased slightly especially in DFS (1.4%) between both groups. In COLOR II trial, patients with stage III operated laparoscopically showed better rate of DFS than the open surgery group with a difference of 12.9% [6]. A recent Spanish population-based study comparing laparoscopic with open surgery that included 1359 patients showed that laparoscopy was an independent factor for better LR and long-term OS rates in rectal cancer [21]. Other published population-based studies with long-term follow-up reported that the laparoscopic approach had better oncological outcomes than the open surgery [22, 23].

Long-term outcomes could be compromised because the perioperative period and the excision of the primary tumor can promote the development of metastases [24]. Surgery could favor the release of tumor cells into the circulation, decrease antiangiogenic factors, increase growth factors, and cause immunosuppression increasing the risk of recurrence [25]. The postoperative immunosuppression is lower after a laparoscopic colorectal resection. The earlier return to normal levels of lymphocytes in the postoperative period of laparoscopic surgery suggests an improvement in restoring immune homeostasis, which could enhance antitumor immune response [26].

This study has some limitations. Firstly, it was a single institutional observational retrospective case matched study on a prospective maintained database. The cohort size and the retrospective design of the study could limit the conclusions drawn from the results. Probably, including other institutions in a multicenter study design would have increased the number of patients analyzed and, therefore, the impact of the study.

Secondly, the whole study period was long, with a variation on the indications for the surgical approach over time. Laparoscopic surgery itself and rectal cancer treatment have evolved during this period. The sample size did not allow stratification by year of treatment, so it could not be incorporated in the propensity score to avoid this bias. Furthermore, the heterogeneity of the cases did not permit an exact matching of two cases operated by open surgery for each case operated by laparoscopy. Some other confounding factors may influence our findings despite adjusted propensity score methodology.

## Conclusion

In conclusion, in unselected patients with mid or low rectal cancer (stages I–III), the laparoscopic resection showed similar long-term oncological outcomes than the open resection. Regardless of the approach, it is essential to respect the embryologic planes during dissection in order to perform oncologically adequate surgery. The differences in the quality of the mesorectum observed could be due to the traction maneuvers during laparoscopic dissection and for this reason did not compromise the oncologic results. Laparoscopic low anterior resection could be considered the technique of choice in rectal cancer although we must wait for the long-term oncological results of high-quality randomized studies to make a definitive statement.
